# Supplementary material for: Knowledge and Expectations of Orthodontic Retention Among Individuals Seeking Orthodontic Treatment in Saudi Arabia: A Cross-Sectional Study
Source: Dent J (Basel). 2026 Jan 4;14(1):21. doi: 10.3390/dj14010021 (PMC12840533; doi:10.3390/dj14010021)
Supplement: Supplementary file 1 [file dentistry-14-00021-s001.zip › File S2. questioner.pdf]

Details of the questioner used.

This study aims to evaluate the knowledge and expectations of orthodontic retention among individuals seeking orthodontic treatment in Saudi Arabia This is a short questionnaire. If you agree to participate, please All it out. Your acceptance to complete the survey will be interpreted as your informed consent to participate.

يهدف هذا البحث لقياس المعرفة والتوقعات بثبات الأسنان بعد التقويم وبإستخدام مثبت الأسنان بعد التقويم بين الأفراد الراغبين في إجراء علاج تقويم الأسنان في المملكة العربية السعودية. هذا استبيان قصير. إذا كنت توافق على المشاركة، يرجى تعبئته. سيتم اعتبار قبولك لإكمال الاستبيان بمثابة موافقة منك على المشاركة في البحث.

1- Do you consent to participate in this study:

هل توافق على المشاركة في هذه الدراسة؟

- Yes / نعم
- No / لا

2- Are you seeking orthodontic treatment for yourself, or are you a parent/guardian of a child under 18 who will undergo orthodontic treatment:

هل□ت مقبل على علاج تقويم الأسنان بنفسك، أم□ك ولي أمر لطفل يقل عمره عن 18 سنة مقبل على علاج تقويم الأسنان؟

- I am seeking orthodontic treatment for myself / □ا مقبل على علاج تقويم الأسنان
- I am a parent/guardian of a child under 18 who is seeking orthodontic treatment / □ا ولي أمر لطفل يقل عمره عن 18 سنة مقبل على علاج تقويم الأسنان

3- Gender / الجنس:

- Male / ذكر
- Female / □ثى

4- Age / العمر:

- 18-24
- 25-34
- 35-44
- 45-54
- +55

5- Educational level / المستوى التعليمي :

- Uneducated / غير متعلم
- High school or lower education / شهادة الث□وية العامة أو اقل
- Bachelor degree / شهادة البكالوريوس
- Postgraduate degree/ شهادة دراسات عليا

6- Do you have any close family members who have undergone orthodontic treatment? / هل لدى أحد من أفراد أسرتك المقربين تجربة سابقة مع تقويم الأسنان؟

- Yes / نعم
- No / لا

7- What is the reason for your orthodontic consultation? / ما سبب زيارتك لعيادة تقويم الأسنان؟

- Self-motivated / رغبة شخصية
- Referral by someone / تحويل من شخص آخر

Knowledge Of Orthodontic Retention Among Individuals Seeking Orthodontic Treatment/ المعرفة بثبات الأسنان بعد التقويم وبإستخدام مثبت الأسنان بعد التقويم

8- Are you aware that appliances are used for retention after orthodontic treatment? / هل تعلم أنه يتم استخدام أدوات/اجهزة لتثبيت الأسنان بعد علاج تقويم الأسنان؟

- Yes / نعم
- No / لا

9- How often do you think such appliances are necessary? / ما مدى اعتقادك بضرورة استخدام مثل هذه الأدوات/الاجهزة؟

- In rare cases / في حالات نادرة
- In some cases / في بعض الحالات
- In most cases / في معظم الحالات
- In all cases / في جميع الحالات

10- In which cases do you consider retention necessary? (Select all that apply)

في أي الحالات التالية تعتقد أن تثبيت الأسنان ضرورياً؟ (اختر كل ما ينطبق)

- After comprehensive orthodontic treatment / بعد العلاج التقويمي الشامل
- After treatment with extractions / بعد العلاج التقويمي مع خلع الأسنان
- After orthodontic treatment during growth / بعد العلاج التقويمي خلال فترة النمو
- After orthodontic treatment in adults / بعد العلاج التقويمي للبالغين
- In all cases / في جميع الحالات

11- Do you believe a perfect treatment result can guarantee stability? / هل تعتقد أن تحقيق نتيجة علاج مثالية يضمن ثبات الأسنان؟

- Yes / نعم
- No / لا

12- Do you think that teeth can also move without orthodontic appliances? / هل تعتقد أن الأسنان يمكن ان تتحرك بدون عمل تقويم للأسنان؟

- Yes / نعم
- No / لا

Expectations Toward Orthodontic Retention / التوقعات نحو تثبيت الأسنان بعد علاج تقويم الأسنان

13- How long do you think the retention phase should be? / برأيك، ما المدة المناسبة لفترة التثبيت بعد علاج تقويم الأسنان؟

- Less than 1 year / اقل من سنة
- 1-3 years / ١-٣ سنوات
- 3-10 years / ٣-١٠ سنوات

- Lifelong / مدى الحياة

14- How important is a stable result for you? / ما مدى أهمية الحصول على نتيجة ثابتة ومستقرة بالنسبة لك؟
- Not important / غير مهم
- Ambivalent / متردد
- Rather important / مهم لحد كبير
- Extremely important / في غاية الأهمية

15- Which type of retention device would you favor? / إي نوع من أجهزة التثبيت تفضل استخدامه بعد علاج تقويم الاسنان؟
- Removable device / جهاز تثبيت متحرك
- Bonded device / جهاز تثبيت ثابت

16- At which interval do you believe recall visits are necessary? / برأيك، ما هو الفاصل الزمني المناسب لزيارات المتابعة بعد علاج تقويم الأسنان؟
- Every 3 months / كل ٣ اشهر
- Every 6 months / كل ٦ اشهر
- Yearly / سنوياً
- Every 2nd year / كل سنتين
- Every 5th year / كل ٥ سنوات

17- Who do you consider responsible for the stability after orthodontic treatment? (Select all that apply) / برأيك، من المسؤول عن الحفاظ على استقرار النتائج بعد علاج تقويم الأسنان؟ (اختر كل ما ينطبق)
- Patient and/or parent / المريض و/أو الوالدين
- General dentist / طبيب الأسنان العام
- Orthodontist / طبيب تقويم الأسنان

18- Do you think it is appropriate to charge for recall visits? / هل تعتقد أنه من المناسب فرض رسوم على زيارات المتابعة بعد علاج تقويم الأسنان؟
- Yes / نعم
- No / لا

<u>Perceived Reliability of Information Sources / مدى الثقة في مصادر المعلومات</u>

19- How confident are you in the accuracy of the information you receive from these sources about orthodontic retainers?
ما مدى ثقتك في دقة المعلومات التي تحصل عليها من هذه المصادر حول مثبتات الأسنان بعد التقويم؟

1 – Very not confident / غير واثق على الإطلاق
2 – Somewhat not confident / غير واثق الى حد ما
3 – Neutral / محايد
4 – Somewhat confident / واثق الى حد ما
5 – Very confident /واثق جدا

- Orthodontists /طبيب تقويم الأسنان
- General dentist / طبيب الأسنان العام

- Friends and Family / الأصدقاء والعائلة

- Online searches (Google, etc.) /البحث عبر الإنترنت (جوجل، إلخ)

- Social media (Instagram,Tiklok, etc.) \ وسائل التواصل الاجتماعي(إنستغرام، تيك توك، إلخ)

- Artificial Intelligence Chatbots (e.g ChatGPT) / روبوتات الدردشة المدعومة بالذكاء الاصطناعي (مثل ChatGPT)
